# Up-Regulation of microRNA* Strands by Their Target Transcripts

**DOI:** 10.3390/ijms140713231

**Published:** 2013-06-26

**Authors:** Sung-Min Kang, Ji-Woong Choi, Su-Hyung Hong, Heon-Jin Lee

**Affiliations:** 1 Department of Oral Microbiology, School of Dentistry, Kyungpook National University, Daegu 700-412, Korea; E-Mails: dkdkdk43@naver.com (S.-M.K.); cjwboyboy@naver.com (J.-W.C.); hongsu@knu.ac.kr (S.-H.H.); 2 Brain Science and Engineering Institute, Kyungpook National University, Daegu 700-412, Korea

**Keywords:** microRNA (miRNA), pre-miRNA, RISC, Drosha, Dicer, Argonaute (Ago)

## Abstract

During microRNA (miRNA) biogenesis, one strand of a 21–23 nucleotide RNA duplex is preferentially selected for entry into an RNA-induced silencing complex (RISC). The other strand, known as the miRNA* species, is typically thought to be degraded. Previous studies have provided miRNA* selection models, but it remains unclear how the dominance of one arm arises during the biogenesis of miRNA. Using miRNA sponge-like methods, we cloned four tandem target sequences (artificial target) of miR-7b* and then measured miR-7b* expression levels after transfection of the artificial target. miR-7b* levels were found to significantly increase after transfection of the artificial target. We postulate that the abundance of target transcripts drives miRNA arm selection.

## 1. Introduction

MicroRNAs (miRNAs) are noncoding 21–23 nucleotide (nt) strands and constitute an evolutionarily conserved class of pleiotropically acting small RNAs. miRNAs usually control posttranscriptional processes, such as, sequence-specific interactions with 3′ untranslated regions (UTRs) of cognate mRNA targets in animals [1]. Nucleotides at positions 2–8 are considered to be important for pairing with target messenger RNAs and are referred to as “seed” sequences [2,3].

A miRNA gene is first transcribed into a primary miRNA (pri-miRNA), then processed into a ~70 nt hairpin precursor miRNA (pre-miRNA) by the RNase III enzyme Drosha and double-stranded RNA-binding domain protein, DGCR8 [4]. Pre-miRNA is then cleaved to generate the ~22 nt miRNA:miRNA* duplex by another RNase III enzyme, Dicer [5]. One strand of the duplex is loaded onto Argonaute (Ago) protein to produce RNA-induced silencing complex (RISC). Furthermore, it has been suggested that, whereas all Ago proteins participate in the stabilization of mature miRNA, only Ago2 (which has endonuclease activity) cleaves the miRNA* strand and activates RISC [6–9]. This suggestion led to the observation that Ago1 facilitated RISC-mediated translational repression and Ago2-RISC led to target mRNA cleavage [8,10]. However, Ago proteins have also been demonstrated to have dual roles, for example, Ago proteins increased the abundance of mature miRNAs, and decreased miRNA expression was observed in a cell line from an Ago2 knockout mouse [11].

The nomenclature originated because one arm, the miRNA, of the RNA duplex preferentially accumulates and the opposite arm, miRNA*, degrades. Another nomenclature often used is miR-3p/miR-5p, which refers to the direction of the mature miRNA strand. 3p and 5p miRNAs usually exhibit partial complementary overlap and have different target genes, despite being produced from the same transcript [12]. However, it remains unclear how dominance of one arm arises during the biogenesis of miRNA. Previous models suggest that the choice of the dominant miRNA arm is based on two mechanisms, that is, on the thermodynamic stability and structural properties of the processed duplex [13,14], or on energy-independent protein-mediated selection by Ago2, an endonuclease that cleaves complementary siRNA strands to facilitate RISC loading of the siRNA strand [6].

However, in a recent study, it was suggested the hairpin arm that makes dominant miRNA differs in different tissues, at different times of development, and between species [15]. In human gastric cancer, miRNA hairpin arm (3p or 5p) selection exhibits different tissue expression preferences in healthy and tumor tissues [16]. Furthermore, some miRNA precursors are processed to produce significant amounts of mature miRNAs from both arms and both miRNAs might regulate target transcripts [17]. These findings suggest the existence of another mechanism for controlling the selection of mature miRNAs.

We expressed an artificial target of miR-7b* that normally presents less than its mature miRNA, miR-7b in order to investigate the effect of the target mRNA on miRNA*. The expression of artificial targets can force the accumulation of miR-7b* rather than miR-7b, which suggests that target abundance might be a critical prerequisite of miRNA* strand stabilization.

## 2. Results and Discussion

**Figure 1:**
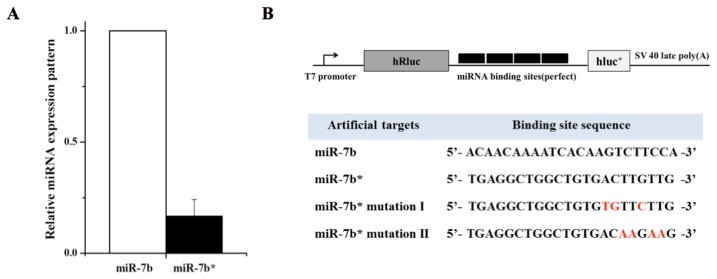

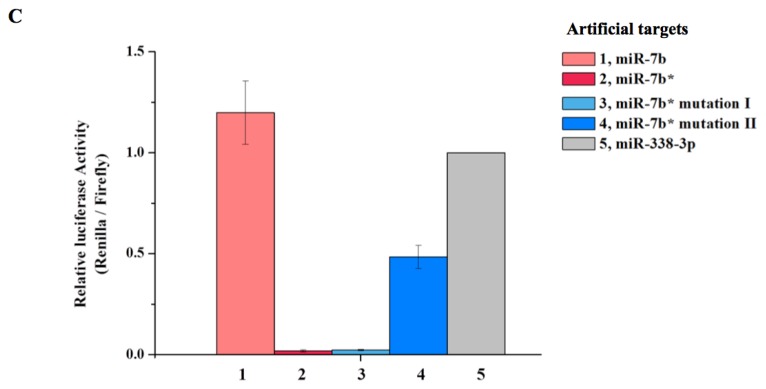
The expression patterns of miR-7b and miR-7b*. (**A**) Basal levels of miR-7b and miR-7b* expression in AtT-20 cells were analyzed by qRT-PCR; (**B**) Design of miRNA artificial targets. *Renilla* (hR*luc*) containing miRNA artificial targets were constructed by inserting multiple miRNA binding sites into the 3′ UTR of a hR*luc* reporter gene driven by a T7 promoter. The figure shows the nucleotide sequence of the miRNA artificial targets: the red letters are a mutated sequence; (**C**) Luciferase assay using the miRNA artificial target reporter constructs. COS-7 cells were co-transfected with the miR-7b* mimic oligonucleiotide and miRNA artificial target (1. miR-7b, 2. miR-7b*, 3. miR-7b* mutation I, 4. miR-7b* mutation II, 5. miR-338-3p). *Renilla* luciferase activity was normalized against *firefly* luciferase activity and fold changes are compared to miR-338-3p. The results shown are the means of three independent transfections (error bars indicate standard deviations).

**Figure 2:**
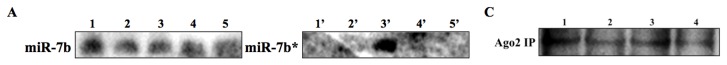

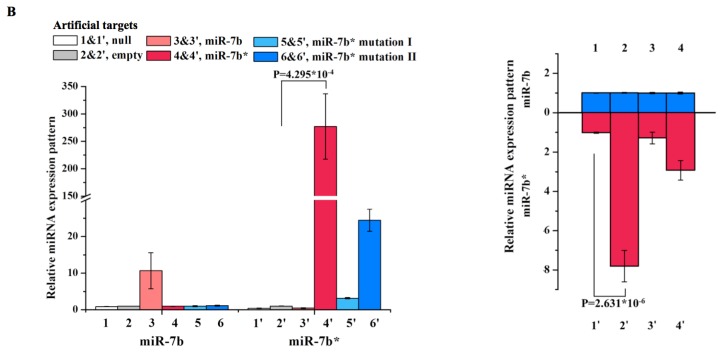
Changed expression levels of miR-7b and miR-7b* after transfection with multi-transcript artificial targets. (**A**) 24 h after transfection of miRNA artificial targets (1 and 1′. null, 2 and 2′. empty vector, 3 and 3′. miR-7b*, 4 and 4′. miR-7b* mutation I, 5 and 5′. miR-7b* mutation II), AtT-20 total RNA was extracted and analyzed by northern blotting using probes specific for miR-7b and miR-7b*, respectively. Lane 3′ shows that miR-7b* expression was significantly increased following transfection of the miR-7b* artificial target. In contrast, miR-7b expression was similar in non-transfected and miR-7b* artificial targets transfected samples; (**B**) Twenty-four hours after various artificial targets (1 and 1′. null, 2 and 2′. empty vector, 3 and 3′. miR-7b, 4 and 4′. miR-7b*, 5 and 5′. miR-7b* mutation I, 6 and 6′. miR-7b* mutation II) were transfected, miR-7b (left) and miR-7b* (right) expression levels were analyzed by qRT-PCR. miRNA expression levels were normalize to U6. Fold changes are expressed *versus* empty target of each (2 or 2′); (**C**) Twenty-four hours after various artificial targets (1 and 1′. empty vector, 2 and 2′. miR-7b*, 3 and 3′. miR-7b* mutation I, 4 and 4′. miR-7b* mutation II) were transfected, AtT-20 lysates were immunoprecipitated (IP) using anti-Ago2 antibody. IPs were analyzed by immunoblotting with the same anti-Ago2 antibody (upper panel). Co-immunoprecipitated RNA was extracted and analyzed by qRT-PCR (lower panel). Fold changes are expressed *versus* the empty target for each (1 or 1′). Error bars in graphs indicate standard deviations; *p*-values are also indicated.

**Figure 3:**
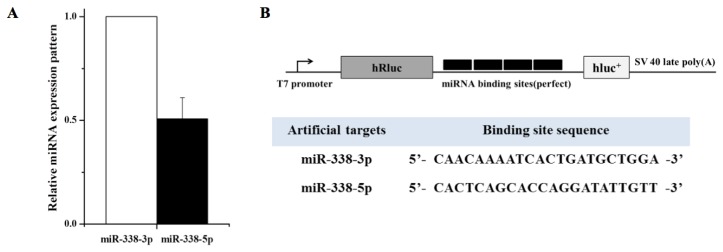

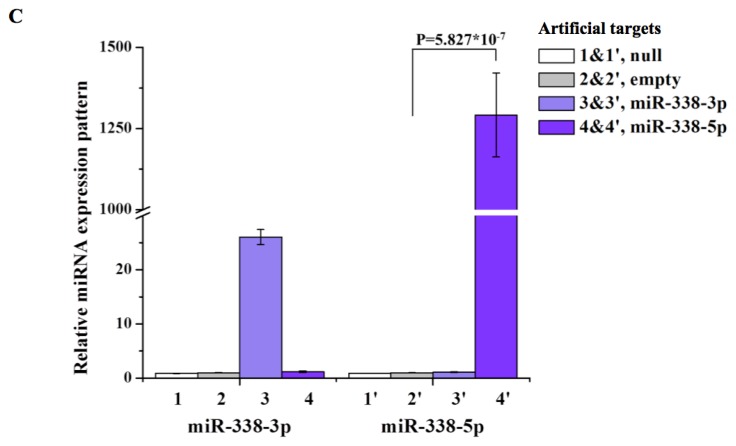
miR-338-3p and miR-338-5p expressions. (**A**) miR-338-3p and miR-338-5p expression were analyzed by qRT-PCR of the total RNA extracted from AtT-20 cells. miR-338-3p levels were about twice as high as miR-338-5p levels in AtT-20 cells; (**B**) Schematic representation of miRNA artificial targets. Construct map of the miR-338-3p and miR-338-5p artificial targets with miR-338-3p and miR-338-5p artificial target sequences below; (**C**) Both miR-338-3p and miR-338-5p expression was up-regulated by each of artificial targets. However, the change of miR-338-3p was not as great as miR-338-5p (miR-338*) by their artificial target. The expression levels of miR-338-5p were significantly elevated by miR-338-5p artificial target transfection but not by transfection of the miR-338-3p artificial target or empty vector (1 and 1′. null, 2 and 2′. empty vector, 3 and 3′. miR-338-3p, 4 and 4′. miR-338-5p). Fold changes are expressed *versus* the empty target for each (2 or 2′). Error bars in graphs indicate standard deviations; *p*-values are also indicated.

The importance of the influences of target mRNA abundance and turnover rates on miRNA activity have been discussed [18,19]. Recently, Chatterjee *et al.* suggested the term “target-mediated miRNA protection (TMMP)”, and showed that target mRNAs in *C. elegans* can protect their cognate miRNAs from degradation *in vivo* [20]. However, little is known about the decay of miRNA*, the other arm of the same hairpin precursor. We hypothesized that target mRNAs of high abundance may drive miRNA arm selection, and in a previous study using miRNA sponge-like methods [21], we cloned multiple target sequences of miR-7b and miR-7b* (miRNA artificial targets; Figure 1B). It has been reported that hyperosmolar stimulation induces miR-7b in the hypothalamus and that the neuronal marker Fos expression is inhibited by miR-7b [22]. Usually, miR-7b is dominant (approximately eight fold higher than miR-7b*) in AtT-20 mouse pituitary cells (Figure 1A). However, in the present study, both qRT-PCR and Northern blotting clearly showed dramatic elevated expression of endogenous miR-7b* by the miR-7b* artificial target. The seed-sequence mutated artificial targets (miR-7b* mutation I and II) reduced the miR-7b* up-regulating effect (Figure 2A,B), suggesting that the up-regulation of miR-7b* occurred in a sequence specific manner (see Figure 1B for artificial and seed mutated targets). Furthermore, luciferase assays showed that miR-7b* mimic oligonucleotide strongly suppressed luciferase activity by binding to its artificial target with a perfect complementary match: this effect was reduced by the seed mutated artificial targets (Figure 1C). We observed the same effects for miR-338-3p (dominant or guide strand) and for miR-338-5p (miR-338* or passenger strand) after artificial targets transfection (Figure 3). Interestingly, when the artificial targets of the dominant strands (miR-7b and miR-338-3p) were transfected, the elevation effect was not as great as that of the artificial target of the non-dominant strands (miR-7b* and miR-338-5p) (Figures 2A,B and 3C). This suggests there might be a certain threshold for this differential regulation or some unknown mechanism that overrides miRNA arm selection.

**Figure 4:**
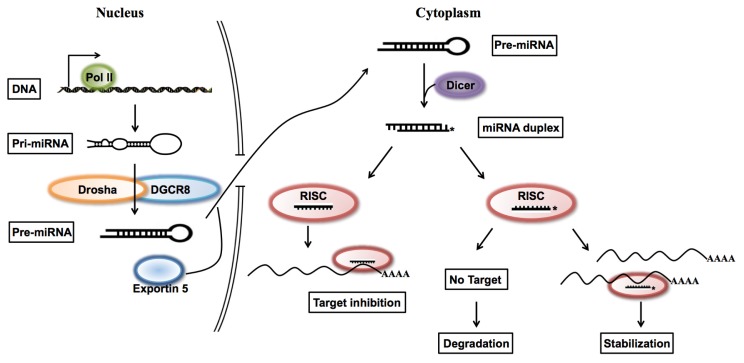
Schematic diagram of miRNA arm selection. Pri-miRNA is transcribed by RNA polymerase II and processed into pre-miRNA by Drosha/DGCR8 in the nucleus. Then pre-miRNA is processed into a duplex form of mRNA that is unwound during RISC assembly in the cytoplasm. Selection of the miRNA* sequence is determined by target transcript abundances. When miRNA* target transcripts are sufficiently abundant the miRNA* sequence is stabilized and acts on target transcripts in the same manner as miRNA.

Since Ago proteins are key players in small RNA-mediated RNA silencing pathways [23], and Ago2 mediates RNA cleavage by harboring RNA catalytic activity in human and mouse [24,25], we performed an Ago2-immunoprecipitation assay to check whether up-regulated miR-7b could interact with Ago2. We found that up-regulated miR-7b* interacted with Ago2 (Figure 2C), which suggests that miR-7b* may have the same inhibitory function as mature miRNAs, similar to the regulatory activity of miRNA* function without degradation [17]. We tried to examine the effect of TMMP on miR-7b* by using endogenous miR-7b* target transcripts, but failed to identify targets of miR-7b experimentally due to the difficulty of predicting miRNA targets. Based on our results, we postulate mature miRNA arm selection is influenced by the abundance of miRNA target transcripts, and that selection may occur as duplex miRNAs, incorporated into the RISC complex (Figure 4), unwind from RISC assembly [26].

## 3. Experimental Section

### 3.1. Cell Culture

COS-7 cells (a monkey kidney fibroblast cell line) were obtained from the Korea Cell Line Bank (Seoul). COS-7 cells were cultured in Dulbecco’s Modified Eagle’s Medium (DMEM/High Glucose) (Hyclone, Logan, UT, USA) containing 10% fetal bovine serum (FBS) (Hyclone, Logan, UT, USA) and 1× antibiotic-antimycotic (GIBCO, Grand Island, NY, USA), and then incubated at 37 °C in a 5% CO_2_ atmosphere. AtT-20 cells (a mouse pituitary tumor cell line) were obtained from the American Type Culture Collection (ATCC, CCL-89), and were grown in Kaighn’s Modification of Ham’s F-12 (F-12K) medium (Hyclone) supplemented with 15% horse serum, 2.5% FBS and 1× antibiotic-antimycotic. These cells were used in the non-luciferase assays.

### 3.2. Construction of Artificial miRNA Target

We annealed, ligated, and cloned oligonucleotides for miRNA binding sites with four tandem target sequences (Figure 1B) into psiCEHCK2 vector (Promega, Madison, WI, USA) downstream of the *Renilla* luciferase (hR*luc*) coding sequence.

### 3.3. Luciferase Assays

COS-7 cells were plated the day before transfection and transfected in triplicate with Lipofectamine 2000 (Invitrogen, Carlsbad, CA, USA) and 800 ng of various artificial target plasmids and 25 nM of miR-7b* mimic oligonucleotide (Bioneer, Daejeon, Korea). All assays were performed 24 h after transfection using the dual luciferase assay (Promega, Madison, WI, USA), according to the manufacturer’s protocol. All experiments were performed in triplicate.

### 3.4. Isolation of miRNA and Quantitative Real-Time PCR (qRT-PCR) Analysis

Total RNAs were extracted from the artificial target transfected AtT-20 cells using QIAzol (Qiagen, Valencia, CA, USA) using a modification of the manufacturer’s instructions, and then treated with DNaseI (Ambion, Foster City, CA, USA). qRT-PCR of miRNAs was conducted on an ABI 7500 real-time PCR system using TaqMan Universal PCR Master Mix, miRNA Expression Assay primer, and probe sets (Applied Biosystems, Foster City, CA, USA). U6 RNA (a small nuclear RNA) was used as an internal cDNA loading control. Threshold cycle times (*C*t) were obtained and relative gene expressions were calculated using the comparative cycle time method.

### 3.5. Immunoprecipitation

For the immunoprecipitation of endogenous Ago2, AtT-20 cells were grown on 10 cm dishes and harvested at 24 h after miRNA transfections. Cells were then incubated with lysis buffer for 20 min on ice, homogenized, and centrifuged at 12,000 rpm for 20 min at 4 °C. Supernatants were incubated with anti-Ago2 antibody (Sigma Aldrich, St. Louis, MO, USA) with constant rotation for one day at 4 °C. Then, 20 μL of protein G Sepharose^®^ beads (Sigma Aldrich, St. Louis, MO, USA) were added and incubated with rotation for 4 h at 4 °C. Beads were then washed three times with lysis buffer.

### 3.6. Isolation of Ago2-Associated miRNA

To measure amounts of RISC-associated miRNAs, cell lysates were prepared from AtT-20 cells after the transfection of miRNA artificial targets. Ago2-miRNA complex was immunoprecipitated from lysates using anti-Ago2 antibody and total RNA was isolated from immunoprecipitates using QIAzol reagent (Qiagen, Valencia, CA, USA). The miRNAs levels were measured by quantitative RT-PCR and normalized against U6 levels in cell lysates.

### 3.7. Northern Blot Analysis for miRNA

Ten μg aliquots of AtT-20 total RNA isolated from AtT-20 cells using QIAzol (Qiagen, Valencia, CA, USA), according to the manufacturer’s instructions, was separated on 15% TBE-urea gels (Invitrogen, Carlsbad, CA, USA) and electro-transferred to Nylon+ membranes (Invitrogen, Carlsbad, CA, USA). Hybridizations were carried out in North2South^®^ hybridization buffer (Invitrogen) at 37 °C using miR-7b* probe (Bioneer, Daejeon, Korea), or at 42 °C using miR-7b LNA (Locked Nucleic Acid) probe (Exiqon, Vedbaek, Denmark).

### 3.8. Statistical Analysis

All data are presented as means ± standard deviations (SD). Significant variation analysis was conducted to calculate the parametric two-tailed non-paired *t*-test. All analyses were performed using Origin 8.0 (OriginLab, Northampton, MA, USA), and *p*-values of ≤0.05 were considered statistically significant.

## 4. Conclusions

Despite extensive study of miRNA, it remains largely unclear how one miRNA arm becomes less dominant (often referred as miRNA*) during the miRNA maturation process. In this study, we introduced an artificial target of miR-7b* in order to check miR-7b* stability. Transfection of the miR-7b* artificial target led to a dramatic up-regulation of miR-7b*, but did not have much effect on miR-7b, the dominant sequence of miR-7b hairpin precursor. A similar phenomenon was observed in miR-338-3p and miR-338-5p (miR-338*). Therefore, we postulate that selection of the miRNA arm might be decided by the mechanism “target-mediated miRNA protection (TMMP)” and TMMP is probably more selective to miRNA* strands.
